# Indian hedgehog gene transfer is a chondrogenic inducer of human mesenchymal stem cells

**DOI:** 10.1186/ar3921

**Published:** 2012-07-20

**Authors:** Andre F Steinert, Manuel Weissenberger, Manuela Kunz, Fabian Gilbert, Steven C Ghivizzani, Sascha Göbel, Franz Jakob, Ulrich Nöth, Maximilian Rudert

**Affiliations:** 1 Department of Orthopaedic Surgery, König-Ludwig-Haus, Center for Musculoskeletal Research, Julius-Maximilians-University, Brettreichstraße 11, Würzburg 97074, Germany; 2 Department of Orthopaedics and Rehabilitation, University of Florida, 1600 SW Archer Road, Gainesville, FL 32610, USA

## Abstract

**Introduction:**

To date, no single most-appropriate factor or delivery method has been identified for the purpose of mesenchymal stem cell (MSC)-based treatment of cartilage injury. Therefore, in this study we tested whether gene delivery of the growth factor Indian hedgehog (IHH) was able to induce chondrogenesis in human primary MSCs, and whether it was possible by such an approach to modulate the appearance of chondrogenic hypertrophy in pellet cultures *in vitro*.

**Methods:**

First-generation adenoviral vectors encoding the cDNA of the human IHH gene were created by cre-lox recombination and used alone or in combination with adenoviral vectors, bone morphogenetic protein-2 (Ad.BMP-2), or transforming growth factor beta-1 (Ad.TGF-β1) to transduce human bone-marrow derived MSCs at 5 × 10^2 ^infectious particles/cell. Thereafter, 3 × 10^5 ^cells were seeded into aggregates and cultured for 3 weeks in serum-free medium, with untransduced or marker gene transduced cultures as controls. Transgene expressions were determined by ELISA, and aggregates were analysed histologically, immunohistochemically, biochemically and by RT-PCR for chondrogenesis and hypertrophy.

**Results:**

IHH, TGF-β1 and BMP-2 genes were equipotent inducers of chondrogenesis in primary MSCs, as evidenced by strong staining for proteoglycans, collagen type II, increased levels of glycosaminoglycan synthesis, and expression of mRNAs associated with chondrogenesis. IHH-modified aggregates, alone or in combination, also showed a tendency to progress towards hypertrophy, as judged by the expression of alkaline phosphatase and stainings for collagen type X and Annexin 5.

**Conclusion:**

As this study provides evidence for chondrogenic induction of MSC aggregates *in vitro *via IHH gene delivery, this technology may be efficiently employed for generating cartilaginous repair tissues *in vivo*.

## Introduction

None of the current approaches to cartilage regeneration, including cell-based therapies, have generated long-lasting hyaline neo-cartilage repair tissue *in vivo *[1]. By means of microfracture treatment, which allows mesenchymal stem cells (MSCs) from the subchondral bone to enter the cartilage defect, MSC-based treatments of cartilage lesions have become part of our daily clinical routine. But administration of *ex vivo *amplified MSCs to repair full-thickness articular cartilage defects in humans has also been introduced into the clinical arena as an alternative to transplantation of autologous chondrocytes [2,3]. However, for such MSC-based therapeutic interventions to be successful, the creation of an appropriate three-dimensional environment that allows induction of chondrogenesis and maintenance of the chondrogenic phenotype *in vivo *is of key importance [1,4]. To achieve this goal probably requires administration of appropriate growth factors at adequate doses, which facilitate the desired aspects of repair. Among these growth factors, the members of the transforming growth factor beta (TGF-β) superfamily have been most extensively analysed, along with many others [5,6]. Furthermore, to overcome the limitations of inadequate delivery of the soluble factors for chondrogenic induction and maintenance, genetic interventions have been explored clinically [7] and extensively in experimental models *in vitro *and *in vivo *[8-10].

Remarkably, despite the fast-growing body of literature on this topic, no single most-appropriate factor and delivery method has been identified for the purpose of hyaline cartilage regeneration that justified approval for testing in humans. One hurdle that might impair stable neo-cartilage formation is the issue of chondrogenic hypertrophy, which has been identified in studies on MSC-mediated chondrogenesis using several bone morphogenetic proteins (BMPs) *in vitro *and *in vivo *[1,8,11-13]. When analysing such models, it appears that BMP-2 and others not only drive MSCs toward chondrogenesis, but also mediate end-stage chondrocyte maturation, hypertrophy and apoptosis, which concurs with endochondral ossification during limb development but is unwanted in cartilage defects [1]. In this context, modulators of chondrogenic hypertrophy in the growth plate such as parathyroid hormone-related peptide (PTHrP) and Indian hedgehog (IHH) [14] appear to be of great interest for the development of MSC-based cartilage repair approaches. Therefore, having extensively explored chondrogenesis in primary MSCs along with the issue of hypertrophy following adenoviral-mediated gene delivery of TGF-β1 and BMP-2 in previous work using pellet cultures *in vitro *[8,11,15], the purpose of this study was to explore the effects of IHH gene transfer in this model.

## Materials and methods

### Generation and propagation of recombinant adenoviral vectors

The full-length cDNA clone of the human IHH gene [GenBank:BC034757, GenBank:BI517875] was released from the carrier pCMV-SPORT6 plasmid (MGC-34815; American Type Culture Collection, Manassas, VA, USA) and the 1.4 kB insert was cloned into Xba/Xho sites of the pAdlox adenoviral shuttle vector [16] according to standard protocols [17]. First-generation, E1-deleted and E3-deleted, serotype 5 adenoviral vectors carrying the cDNAs for human IHH were constructed by cre-lox recombination as described earlier [16] and were designated Ad.IHH. The vectors encoding GFP from jellyfish, TGF-β1 and BMP-2 were generated, amplified, purified and used as described earlier [11,16,18]. Virus stock titres were determined by optical density at 260 nm and standard plaque assay, and ranged between 10^12 ^and 10^13 ^particles/ml.

### Transduction and culture of human marrow-derived MSCs

Human bone marrow-derived MSCs were isolated from marrow reamings of femurs from five patients, aged 48 to 62 years (mean age 54 years), undergoing total hip replacement surgery after informed consent and as approved by the institutional review board of the University of Würzburg as described earlier [11,19]. Briefly, the collected cells were spun, resuspended and placed in tissue culture using complete DMEM containing 10% foetal bovine serum and 1% penicillin/streptomycin, 1 ng/ml fibroblast growth factor-2 (all Invitrogen GmbH, Darmstadt, Germany). At confluency, cells were trypsinised, counted, replated and transduced at 5 × 10^2 ^infectious particles/cell for each vector for 2 hours. Marker gene controls were also maintained along with cultures that remained uninfected and received, or not, supplementation with recombinant 10 ng/ml TGF-β1 protein (R&D Systems, Minneapolis, MN, USA).

### Pellet culture and assessment of transgene expression

Following infection, MSCs were placed in pellet cultures as previously described [11]. Briefly, MSCs were suspended in serum-free DMEM containing 1 mM pyruvate, 1% ITS+ Premix, 37.5 mg/ml ascorbate-2-phosphate and 10^-7 ^M dexamethasone (all Sigma, St Louis, MO, USA), and then 300 μl aliquots (3 × 10^5 ^cells) were distributed to a polypropylene, V-bottomed 96-well plate (Corning, Corning, NY, USA) to promote aggregate formation. As already mentioned, control aggregates were maintained that remained uninfected and received, or not, 10 ng/ml TGF-β1 recombinant protein (all R&D Systems). Pellets formed within 24 hours and cultures were maintained at 37°C, 5% CO_2 _with changes of fresh media being performed every 2 to 3 days until harvest at various time points for further analyses. Twenty-four-hour-conditioned media were collected at several time points and assayed for TGF-β1, BMP-2 (R&D Systems) and IHH (Cusabio Biotech Co. Ltd, Newark, DE, USA) expression using commercially available ELISA kits.

### Osteogenic and adipogenic differentiation

For confirmation of the multilineage differentiation plasticity, selected MSC preparations were additionally tested for their capacity to differentiate along osteogenic or adipogenic lineages as described earlier [20]. MSC monolayer cultures were therefore seeded at a density of 1 × 10^5 ^cells/cm^2 ^in four-well chamber slides (Nunc, Wiesbaden, Germany). For osteogenic induction, media were supplemented with 10^-7 ^M dexamethasone, 50 μg/ml ascorbate, 10 mM β-glycerophosphate, and 25 ng/ml recombinant human BMP-2 (R&D Systems). Adipogenesis was induced on separate monolayer cultures by supplementing the media with 1 μM dexamethasone, 1 μg/ml insulin, 0.5 mM 3-isobutyl-1-methylxanthine and 100 μM indomethacin (all Sigma). Control cultures without osteogenic or adipogenic supplements were also maintained. After 3 weeks, cultures were fixed and osteogenic cultures were stained histochemically for alkaline phosphatase (ALP; Sigma) or for matrix mineralisation using Alizarin Red (Sigma), while adipogenic cultures were stained with Oil Red O for the detection of lipid droplets as previously reported [20].

### Biochemical assays

Biochemical analyses for assessment of cell proliferation, glycosaminoglycan (GAG) synthesis and ALP activity were performed as described previously and as directed by the respective supplier [11]. Briefly, to evaluate cell proliferation a quantitative detection of ATP was performed using the CellTiter-Glo^® ^Luminescent Cell Viability Assay (Promega, Madison, WI, USA). GAG contents were measured following papain digestion (1 μg/ml; Sigma) by reaction with 1,9-dimethylmethylene blue using the Blyscan™ Sulfated Glycosaminoglycan Assay (Biocolor Ltd, Newtownabbey, Northern Ireland). ALP activity in aggregates was analysed using a kit (Sigma) via absorbance at 405 nm by the conversion of *p*-nitrophenyl phosphate to *p*-nitrophenol and inorganic phosphate in an ELISA reader, using a standard curve. Values of the GAG and ALP assays were normalised to DNA content, as determined by the Quant-iT™ PicoGreen^® ^kit (Invitrogen).

### Histology and immunohistochemistry

Histological analyses were performed on aggregates fixed in 4% paraformaldehyde, following dehydration, paraffin embedding, sectioning and staining with H & E and Alcian Blue (all Sigma) as outlined previously [11]. ALP staining was performed using a histochemical ALP staining kit according to the manufacturer's instructions (Sigma). Alternate sections were subjected to immunohistochemistry using pre-digestions and antibody treatments as follows: collagen (COL) type II - pepsin (1 mg/ml; Sigma)/monoclonal anti-COL type II antibodies (Acris Antibodies GmbH, Hiddenhausen, Germany); chondroitin-4-sulphate (CS4) - chondroitinase ABC (5 U/ml; Sigma)/polyclonal anti-CS4 antibodies (Millipore GmbH, Schwalbach, Germany); and COL type × - 0.25% trypsin (Sigma)/polyclonal anti-COL type X antibodies (Calbiochem, Bad Soden, Germany). Immunostainings were visualised by treatment with Advance™ HRP link and Advance™ HRP enzyme (Dako, Hamburg, Germany), followed by diaminobenzidine staining (DAB Kit; Sigma). The slides were finally counterstained with hemalaun (Merck, Darmstadt, Germany). For all immunohistochemical analyses, controls were performed with non-immune IgG (Sigma) instead of the primary antibodies.

### Annexin 5 assay

Annexin 5 expression was assessed as a marker of hypertrophy and apoptosis as described previously [11], and as directed by the supplier (Sigma). Briefly, the test uses double-labelling with the red fluorochome Cy3.18/Annexin 5-Cy3 that binds to early apoptotic cells and conversion of 6-carboxyfluorescein diacetate (nonfluorescent) to 6-carboxyfluorescein (green fluorescent) by living cells. Following incubation with double-labelling staining solution for 10 minutes, aggregates were washed, fixed in 4% paraformaldehyde, and processed to paraffin sections at 4 μm. Evaluation of living and apoptotic cells was performed on representative sections using a fluorescence microscope and the appropriate green and red filters.

### RNA extraction and RT-PCR analyses

On days 3, 7, 14 and 21 RNA was extracted from six to 10 MSC aggregates per group and donors using 1 ml Trizol reagent (Invitrogen) and an additional purification step with separation columns including a DNase treatment according to the manufacturer's instructions (NucleoSpin RNA II Kit; Macherey-Nagel GmbH, Düren, Germany). RNA from pellets of each condition (2 μg each group) was used for reverse transcription using random hexamer primers and BioScript reverse transcriptase (Bioline GmbH, Luckenwalde, Germany). Equal amounts (100 ng) of each cDNA were used as templates for PCR amplification in a 30 μl reaction volume using MangoTaq DNA Polymerase Taq (Bioline GmbH) and 5 pmol of gene-specific primers as listed in Table 1, with elongation factor-1α (EF-1α) serving as the housekeeping gene and internal control. The RT-PCR products were run on 1.5% agarose gels containing 0.1 mg/ml ethidium bromide, and were visualised using the Bio Profile software (LTF, Wasserburg, Germany).

**Table 1:** Primer details used for RT-PCR analyses

Gene	RT-PCR primer sequences (5' to 3')	Annealing temperature (°C)	Product size (base pairs)	Cycles
Chondrogenic markers				
COL type II	Sense: TTTCCCAGGTCAAGATGGTC	58.0	374	35
	Antisense: CTTCAGCACCTGTC CACCA			
AGN	Sense: TGAGGAGGGCTGGAACAAGTACC	54.0	392	30
	Antisense: GGAGGTGGTAATTGCAGGGAACA			
COMP	Sense: CAGGACGACTTTGATGCAGA	54.0	312	32
	Antisense: AAGCTGGAGCTGTCTGGTA			
FMD	Sense: CTTACCCCTATGGGGTGGAT	54.0	389	35
	Antisense: GTACATGGCCGTGAGGAAGT			
SOX9	Sense: ATCTGAAGAAGGAGAGCGAG	58.0	263	35
	Antisense: TCAGAAGTCTCCAGAGCTTG			
Hypertrophy and osteogenic markers				
COL type X	Sense: CCCTTTTTGCTGCTAGTATCC	54.0	468	25
	Antisense: CTGTTGTCCAGGTTTTCCTGGCAC			
OP	Sense: ACGCCGACCAAGGAAAACTC	51.0	483	35
	Antisense: GTCCATAAACCACACTATCACCTCG			
COL type I	Sense: GGACACAATGGATTGCAAGG	54.0	461	32
	Antisense: TAACCACTGCTCCACTCTGG			
OC	Sense: ATGAGAGCCCTCACACTCCTC	59.0	387	35
	Antisense: GCCGTAGAAGCGCCGATAGGC			
ALP	Sense: TGGAGCTTCAGAAGCTCAACACCA	51.0	454	35
	Antisense: TCTCGTTGTCTGAGTACCAGTCC			
Internal control				
EF-1α	Sense: AGGTGATTATCCTGAACCATCC	54.0	234	25
	Antisense: AAAGGTGGATAGTCTGAGAAGC			

AGN, aggrecan core protein; ALP, alkaline phosphatase; COL, collagen; COMP, cartilage oligomeric matrix protein; EF-1α, elongation factor 1α; FMD, fibromodulin; OC, osteocalcin; OP, osteopontin; SOX9, sex determining region Y-box 9.

For semi-quantitative PCR analyses, equal amounts (100 ng) of each cDNA were used as templates for amplification in a 30 μl reaction volume using MangoTaq DNA Polymerase Taq (Bioline GmbH) and 5 pmol of gene-specific primers, which were used to detect mRNA transcripts characteristic of chondrogenic, hypertrophic or osteogenic differentiation states. The sequences, annealing temperatures and product sizes of forward and reverse primers used for COL type II, aggrecan core protein (AGN), cartilage oligomeric matrix protein (COMP), fibromodulin, sex determining region Y-box 9 (SOX9), COL type I, COL type X, osteopontin (OP), and osteocalcin are listed in Table 1, with EF-1α serving as the housekeeping gene and internal control. The RT-PCR products were electrophoretically separated on 1.5% agarose gels containing 0.1 mg/ml ethidium bromide and visualised using Bio Profile software (LTF), allowing correlation between EF-1α signals and cycle number for each sample. The densities of the PCR bands were analysed with the Bio 1D/Capt MW software (LTF), and the mean ratio (fold change) and standard deviation, normalised to expression of the EF-1α housekeeping gene, were calculated from three bands (one per patient).

### Statistical analysis

Data from the ELISA, ATP, GAG, DNA, ALP and PCR analyses were expressed as mean values ± standard deviation, with each experiment being performed in triplicate or quadruplicate (*n *= 3 to 4), and were repeated on at least three to five individual marrow preparations from different patients (*n *= 3 to 5), as indicated in the respective experiments. Numerical data for ATP, GAG, DNA, and ALP were subjected to variance analysis (one-factor or two-factor analysis of variance), and statistical significance was determined by Student's *t *test with *P *< 0.05 considered significant.

## Results

### Multilineage differentiation

**Figure 1:**
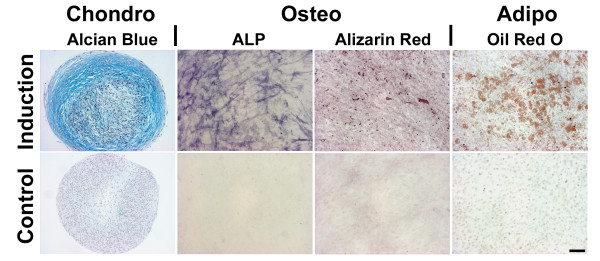
**Multilineage differentiation assays of bone marrow-derived mesenchymal stem cells**. After 3 weeks of pellet culture, mesenchymal stem cells (MSCs) maintained in the presence of transforming growth factor beta (TGF-β1) revealed strong chondrogenic induction as evidenced by strong staining with Alcian Blue, while controls without TGF-β1 were not chondrogenic (left panels). Monolayer cultures of MSCs responded equivalently to culture with osteogenic medium for 2 weeks in terms of strong staining for alkaline phosphatase (ALP) and matrix mineralisation with Alizarin Red as opposed to the weak staining revealed by the negative controls (middle panels). Adipogenesis was observed after 2 weeks of monolayer culture in adipogenic media by staining with Oil Red O for lipid droplets, while only negative staining was detected in the respective control cultures (right panels).

Pellet cultures of MSCs derived from bone marrow underwent chondrogenic differentiation in the presence, but not in the absence, of TGF-β1 as judged by strong metachromatic staining of proteoglycans with Alcian blue (Figure 1, left panels). Monolayer cultures of MSCs responded equivalently to osteogenic or adipogenic medium in terms of strong staining for ALP, mineralisation with Alizarin Red (Figure 1, middle panels), or staining with Oil Red O for lipid droplets (Figure 1, right panels), while only weak staining was observed in the respective control cultures.

### Transgene expression of genetically modified pellet cultures

**Figure 2:**
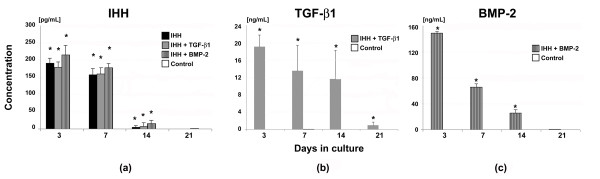
**Transgene expression of mesenchymal stem cell aggregates following adenoviral Indian hedgehog gene transfer**. Primary mesenchymal stem cells were modified with Ad.GFP (controls), Ad.IHH alone (IHH), or in combination with Ad.TGF-β1 (IHH + TGF-β1) or Ad.BMP-2 (IHH + BMP-2), seeded into pellets and analysed during a 21-day time course. Values represent levels of **(a) **IHH, **(b) **TGF-β1, or **(c) **BMP-2 transgene product in the conditioned media by the respective groups over 24 hours at the time points indicated. Mean ± standard deviation from three aggregates per condition and marrow preparation from three marrow preparations from different patients is shown. *Significant differences (*P *< 0.05) compared with controls. Ad., adenoviral vector; BMP, bone morphogenetic protein; IHH, Indian hedgehog; TGF-β, transforming growth factor beta.

Pellet cultures modified with adenoviral vectors encoding IHH alone or together with TGF-β1 or BMP-2 generated high levels of transgene product at day 3 of culture, with dose ranges of 150 to 250 pg/ml for IHH (Figure 2a), 15 to 20 ng ng/ml for TGF-β1 (Figure 2b) and 120 to 160 ng/ml for BMP-2 (Figure 2c), and declining values over time as evidenced by ELISA. This corresponds to previous findings using adenoviral vectors alone [8,11,15] or in combination [11]. The respective values in the marker gene controls (Figure 2a to 2c) were persistently very low (< 10 pg/ml), and were equivalent to the levels observed in the naïve controls (data not shown).

### Histological and immunohistochemical analyses of chondrogenesis

**Figure 3:**
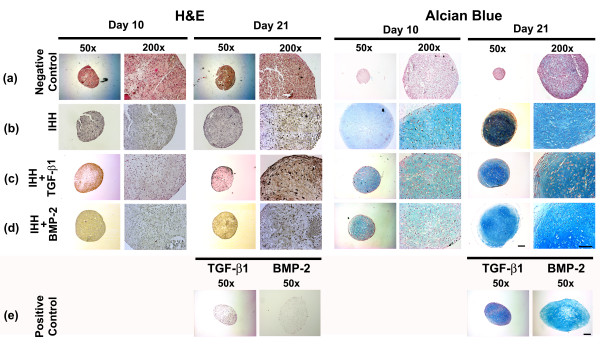
**Histological assessment of chondrogenesis in mesenchymal stem cell aggregates after adenoviral Indian hedgehog gene transfer**. Following genetic modification with **(a) **Ad.GFP (negative control), **(b) **Ad.IHH (IHH), **(c) **Ad.IHH and Ad.TGF-β1 (IHH + TGF-β1), **(d) **Ad.IHH and Ad.BMP-2 (IHH + BMP-2), or **(e) **Ad.TGF-β1 or Ad.BMP-2 only (positive control; TGF-β1 or BMP-2) at 5 × 10^2 ^virus particles/cells, mesenchymal stem cells were maintained as pellets for 3 weeks until aggregates were harvested and processed. Representative sections after 10 and 21 days are shown. Left: H & E staining for evaluation of cellularity and cell morphology. Right: Metachromatic staining with Alcian Blue for detection of matrix proteoglycans. Panels reproduced at low (50×; bar = 200 μm) and high (200×; bar = 50 μm) magnification as indicated. Ad., adenoviral vector; BMP, bone morphogenetic protein; IHH, Indian hedgehog; TGF-β, transforming growth factor beta.

Transduction of MSCs with Ad.IHH (IHH; Figure 3b), Ad.IHH and Ad.TGF-β1 (IHH + TGF-β1; Figure 3c), or Ad.IHH and Ad.BMP-2 (IHH + BMP-2; Figure 3d) induced a significant chondrogenic response in the respective pellet cultures over time compared with the Ad.GFP cultures (positive control; Figure 3a), which were not chondrogenic. Cellularity of the respective groups after 10 and 21 days is shown by H & E staining of representative aggregate sections in Figure 3a, b (left panels). Chondrogenic differentiation was evidenced by strong metachromatic staining for proteoglycans with Alcian Blue (Figure 3a to 3d, right panels) in the extracellular matrix of the IHH, IHH + TGF-β1, and IHH + BMP-2 pellets compared with the marker gene negative controls. Only the comparative positive control groups TGF-β1 or BMP-2 also revealed a chondrogenic phenotype for both stains (Figure 3e).

**Figure 4:**
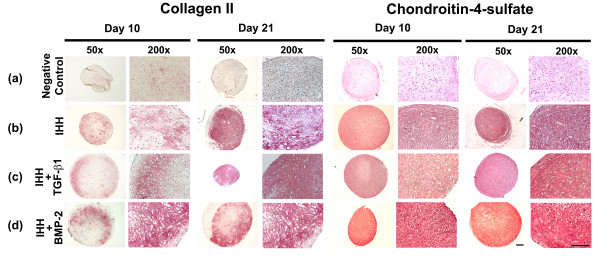
**Immunohistochemical analyses for cartilage matrix proteins of mesenchymal stem cell aggregate culture after chondrogenic induction**. Cells were infected with **(b) **Ad.IHH, **(c) **Ad.IHH + Ad.TGF-β1, **(d) **Ad.IHH + Ad.BMP-2, or **(a) **Ad.GFP (control) at 5 × 10^2 ^virus particles/cells, and immunohistochemical stainings were performed on culture days 10 and 21 or representative sections for collagen type II (left panels) and chondroitin-4-sulfate (right panels). Regions of positive immunostaining appear red. Panels are reproduced at low (50×; bar = 200 μm) and high (200×; bar = 50 μm) magnification as indicated. Ad., adenoviral vector; BMP, bone morphogenetic protein; IHH, Indian hedgehog; TGF-β, transforming growth factor beta.

Correspondingly, immunohistochemistry for cartilage matrix proteins COL type II (Figure 4a to 4d, left panels) and CS4 (Figure 4a to 4d, right panels) showed a strong production of these cartilage matrix proteins at days 10 and 21 of culture in the IHH (Figure 4b), IHH + TGF-β1 (Figure 4c), and IHH + BMP-2 aggregates (Figure 4d) relative to the Ad.GFP controls (Figure 4a), which showed only very low levels of staining for both markers. Clear-cut differences in the chondrogenic appearance of the immunophenotype between the IHH, IHH + TGF-β1, and IHH + BMP-2 aggregates were not observed (Figure 4b to 4d).

Aggregates transduced with TGF-β1 or BMP-2 only, as well as untransduced pellets maintained in the presence of 10 ng/ml recombinant TGF-β1 protein, were all also chondrogenic, while control cultures without gene or growth factor supplementation were nonchondrogenic, by means of positive staining for Alcian Blue (Figure 1, left panels) and immunohistochemistry (data not shown) for COL type II and CS4. This finding corresponds with findings published extensively in previous work [8,11,15].

### Hypertrophic differentiation and apoptosis

**Figure 5:**
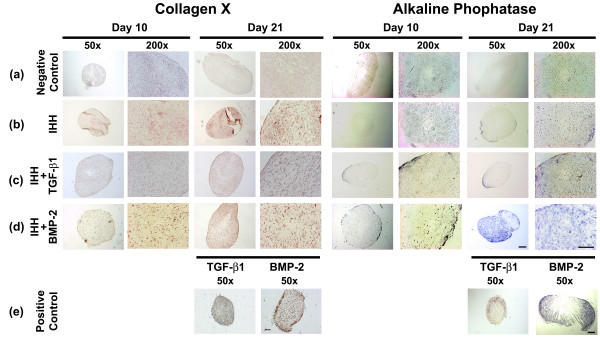
**Histological and immunohistochemical analyses for hypertrophy of mesenchymal stem cell aggregate culture after chondrogenic induction**. After genetic modification with **(a) **Ad.GFP (negative control), **(b) **Ad.IHH (IHH), **(c) **Ad.IHH + Ad.TGF-β1 (IHH + TGF-β1), **(d) **Ad.IHH + Ad.BMP-2 (IHH + BMP-2), or **(e) **Ad.TGF-β1 or Ad.BMP-2 only (positive control; TGF-β1 or BMP-2) at 5 × 10^2 ^virus particles/cells, the aggregate cultures were processed and stained at culture days 10 and 21 for collagen type × (left panels) and alkaline phosphatase (right panels). Regions of positive staining appear brown (left panels) or blue (right panels). Panels are reproduced at low (50×; bar = 200 μm) and high (200×; bar = 50 μm) magnification as indicated. Ad., adenoviral vector; BMP, bone morphogenetic protein; IHH, Indian hedgehog; TGF-β, transforming growth factor beta.

Immunohistochemistry for COL type × (Figure 5a to 5d, left panels) and staining for ALP (Figure 5a to 5d, right panels) were performed on representative aggregate sections after days 10 and 21 of culture and were used as markers for chondrocyte hypertrophy. Negative controls (Ad.GFP) showed no detectable staining for COL type × (Figure 5a, left panels) and ALP (Figure 5a, right panels). In contrast, stronger staining for COL type × (left panels) and ALP (right panels) was found in all chondrogenic groups of aggregates tested (Figure 5b to 5e). Notably, the strongest staining of the matrix for Col type X was seen in the IHH only group (Figure 5b, left panels), while the most extensive staining for ALP was seen the IHH + BMP-2 group and the BMP-2-only positive controls after 21 days of culture (Figure 5d, e, right panels).

**Figure 6:**
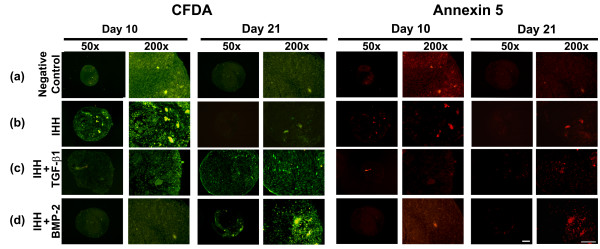
**Analyses for cell viability and apoptosis within mesenchymal stem cell pellets after chondrogenic induction**. Following genetic modification with **(b) **Ad.IHH (IHH), **(c) **Ad.IHH + Ad.TGF-β1 (IHH + TGF-β1), **(d) **Ad.IHH + Ad.BMP-2 (IHH + BMP-2) and **(a) **Ad.GFP (negative control) at 5 × 10^2 ^virus particles/cells, cultures were double-stained with 6-carboxyfluorescein diacetate (CFDA) (left panels) and Annexin 5 (right panels) at days 10 and 21 of culture. Representative fluorescence microscopy images are shown. Note that living cells are stained green with CFDA, late apoptotic cells red with Annexin 5-Cy3, while early apoptotic cells stained for both CFDA and Annexin 5. Panels are reproduced at low (50× bar = 200 μm) or high (200×; bar = 50 μm) magnification as indicated. Ad., adenoviral vector; BMP, bone morphogenetic protein; IHH, Indian hedgehog; TGF-β, transforming growth factor beta.

For evaluation of cell viability and apoptosis (Figure 6) following adenoviral gene transfer using IHH gene transfer alone (Figure 6b) or in combination with TGF-β1 (Figure 6c) or BMP-2 (Figure 6d) compared with untransduced controls (Figure 6a), aggregate cultures were examined using double fluorescence staining with Annexin 5-Cy3/6-carboxyfluorescein diacetate. All groups revealed equivalent high levels of green fluorescence (viable cells) after 10 and 21 days of culture (left panels). In the apoptosis detection assay (Figure 6, right panels), only very few cells were Annexin 5-positive (red fluorescent) in the control group (Figure 6a), as well as in the IHH group (Figure 6b) or the IHH + TGF-β1 group (Figure 6c), while many Annexin 5-positive cells were found in the IHH + BMP-2 group (Figure 6d).

### Biochemical analyses of cell proliferation, GAG content and ALP activity

**Figure 7:**
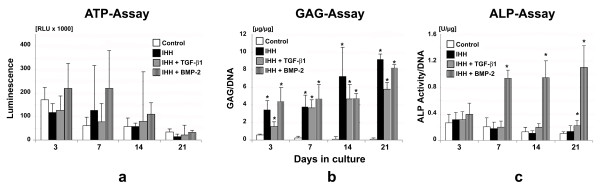
**Biochemical analyses of mesenchymal stem cell aggregates following chondrogenic induction**. Primary mesenchymal stem cells were infected with Ad.GFP (controls), Ad.IHH alone (IHH), or in combination with TGF-β1 (IHH + TGF-β1) or BMP-2 (IHH + BMP-2), placed into aggregate cultures and assayed biochemically over a 3-week time course. **(a) **Cell proliferation was evaluated using the ATP assay, and **(b) **glycosaminoglycan (GAG) content and **(c) **relative alkaline phosphatase (ALP) activity normalised to DNA are shown for quantification of cartilage matrix production and hypertrophy, respectively. Bars represent mean ± standard deviation from three pellets per group and patient, assessed on five marrow preparations from different patients. *Significant difference (*P *< 0.05) compared with marker gene-transduced controls. Ad., adenoviral vector; BMP, bone morphogenetic protein; IHH, Indian hedgehog; TGF-β, transforming growth factor beta.

As primary MSCs were shown to be amenable to adenoviral gene transfer, and to undergo chondrogenesis upon stimulation with IHH alone or in combination with TGF-β1 or BMP-2, we examined the effects of these treatments on cell proliferation using the ATP assay compared with marker gene transduced controls (Figure 7a). Initially, at days 3 and 7, cell proliferation rates in MSC aggregates were high, and they declined over time thereafter, without significant differences between all groups tested (Figure 7a).

Quantitative assessment of GAG synthesis revealed significantly enhanced GAG levels of all treatment groups that received IHH relative to controls (Figure 7b), while major differences in the pattern of GAG synthesis over time among the chondrogenic groups IHH, IHH + TGF-β1, or IHH + BMP-2 could not be resolved (Figure 7b).

Chondrogenic hypertrophy was quantified using ALP activity (Figure 7c), which was found significantly elevated at days 7, 14 and 21 of culture in the IHH + BMP-2 modified aggregates compared with all other groups, whereas significantly higher values in the IHH + TGF-β1 transduced cultures compared with the GFP controls could only be resolved at day 21 (Figure 7c).

### Evaluation of chondrogenic marker gene expression over time

**Figure 8:**
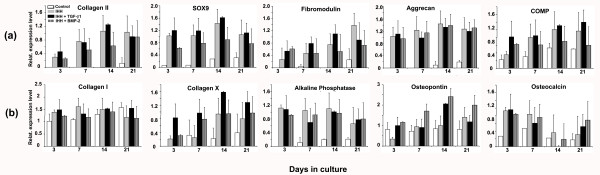
**Time course of gene expression assessed by semiquantitative RT-PCR of mesenchymal stem cell pellet cultures**. Pellets transduced with Ad.GFP (control), Ad.IHH alone (IHH), or Ad.IHH in combination with Ad.TGF-β1 (IHH + TGF-β1) or BMP-2 (IHH + BMP-2) were analysed over time. **(a) **Expressions for the chondrogenic mRNAs collagen (COL) type II, aggrecan core protein, cartilage oligomeric matrix protein (COMP), fibromodulin, or sex determining region Y-box 9 (SOX9) at days 3, 7, 14, or 21 of culture are shown, respectively. **(b) **Expressions of mRNAs associated with chondrogenic hypertrophy and osteogenesis of COL type I, COL type X, alkaline phosphatase, osteopontin, or osteocalcin at days 3, 7, 14, or 21 of culture are shown, respectively. Expressions of elongation factor-1α served as the housekeeping gene and internal control. Primer details are shown in Table 1. Ad., adenoviral vector; BMP, bone morphogenetic protein; IHH, Indian hedgehog; TGF-β, transforming growth factor beta.

To explore the effects of IHH gene delivery alone or in combination with TGF-β1 or BMP-2 on chondrogenesis and hypertrophy compared with controls, we examined the time course for expression of genes associated with chondrogenesis and hypertrophic maturation using semi-quantitative RT-PCR (Figure 8).

Expressions of mRNAs from the chondrogenic marker genes COL type II, AGN, COMP, fibromodulin and SOX9 were upregulated in the IHH, IHH + TGF-β1, and IHH + BMP-2 groups compared with controls (Ad.GFP), which were not chondrogenic and expressed only very low levels of the chondrogenic genes COL type II, AGN, fibromodulin and SOX9 (Figure 8a). Although expressed at lower levels compared with the chondrogenic groups IHH, IHH + TGF-β1, and IHH + BMP-2, above-baseline levels of expressions were found for COMP in the Ad.GFP controls (Figure 8a). Clear-cut differences in the expression pattern of the chondrogenic markers between the chondrogenic groups IHH, IHH + TGF-β1, or IHH + BMP-2 could not be resolved (Figure 8a).

Similarly, temporal expression profiles of mRNAs associated with chondrogenic hypertrophy and osteogenesis revealed elevated levels in all groups and time points tested for COL type I, COL type X, ALP, OP, and osteocalcin at days 3, 7, 14 and 21 of culture (Figure 8b). While the chondrogenic groups IHH, IHH + TGF-β1, and IHH + BMP-2 showed markedly increased mRNA levels for COL type X and ALP compared with the marker gene controls, expressions for COL type I, OP and osteocalcin appeared not to be strongly regulated by the different groups (Figure 8b). Again, major differences between the different chondrogenic groups IHH, IHH + TGF-β1, or IHH + BMP-2 could not be detected.

## Discussion

IHH belongs to the hedgehog protein family, which also includes sonic hedgehog (SHH) and desert hedgehog [21]. Following binding to their cell surface receptors patched-1 and smoothened, hedgehog proteins signal via members of the Gli family of transcription factors (Gli1, Gli2 and Gli3) at the end of the pathway, which are essential regulators of cell fate and patterning in the developing skeleton [22]. In this work, we used the pellet culture system to analyse whether adenoviral delivery of IHH can lead to chondrogenesis of primary MSCs *in vitro*, and to evaluate the extent of hypertrophy alone and together with TGF-β1 or BMP-2.

Delivery of IHH via adenoviral vectors alone or in combination led to reproducible chondrogenesis in human MSC pellet cultures, as shown qualitatively by staining with Alcian Blue, COL type II and CS4 (Figures 3 and 4) and quantitatively using the GAG assay (Figure 7b). This corresponds to findings in the same model, when adenoviral delivery of TGF-β1 [8,15], BMP-4 [11] or BMP-2 [8,11,15] was used. However, combined delivery of IHH together with TGF-β1 or BMP-2 was not able to elicit synergistic effects with respect to the extent of chondrogenesis compared with single IHH delivery (Figures 3, 4 and 7b). This is in agreement with findings of combined gene delivery of TGF-β1 and BMP-2 in the same setup, where additive effects were also absent [23]. In contrast, synergistic effects on transgene expression and chondrogenesis were reported, when TGF-β1 or BMP-2 were administered together with insulin-like growth factor-1 (IGF-1) [23], indicating that the choice of transgenes plays pivotal roles to induce additive chondrogenic effects. Furthermore, increased dosing of adenoviral vectors was shown to be detrimental for chondrogenesis in this model [23], which was the main reason for not increasing the adenoviral dosage in this study. Notably, pellet cultures of all groups decreased levels of cell proliferation over time, with the highest levels being seen in the IHH + BMP-2 groups (Figure 7a). This also corresponds to previous findings, where high levels of cell proliferation and metabolic activity were seen in BMP-4-modified and BMP-2-modified aggregates throughout the 3 weeks of culture [11]. Furthermore, signs of chondrogenic hypertrophy were noticed in MSC pellet cultures transduced with IHH alone and together with TGF-β1, but were most abundant in the IHH + BMP-2 group, as assessed by expression for ALP, COL type X and Annexin 5 (Figures 5 and 6). This also concurs with previous results, where BMP-2-infected pellets revealed higher levels of hypertrophy compared with TGF-β1 [23] or BMP-4 [11].

The data on expression of marker genes for chondrogenesis and hypertrophy (Figure 8) are in general agreement with the biochemical (Figure 7) and histological observations (Figures 3 to 6), showing strong presence of chondrogenic transcripts in aggregates after IHH stimulation, such as COL type II, AGN, COMP and SOX9. Levels of mRNAs encoding the hypertrophy-associated genes COL type X and OP were also present in all IHH-modified groups, compared with controls (Figure 8), and are in general agreement with previous observations where gene delivery of BMP-2 revealed high levels of hypertrophy [11,23].

Our results are in agreement with those of Kellner and colleagues, who used recombinant hedgehog proteins for generation of chondrocyte-based cartilage constructs *in vitro *[24]. In their study, recombinant IHH was as effective as the protein family member SHH to increase levels of GAG synthesis, when proteins were used in dipalmitoylated form at 1,000 ng/ml [24]. In our study, however, IHH was detected at much lower concentration (Figure 2a) but was also very efficient in stimulating chondrocyte matrix synthesis by MSC aggregates (Figures 3, 4 and 7b), which is probably due to the efficient pericellular distribution of the IHH transgene product following adenoviral transduction.

Our data also correspond to positive effects of hedgehog proteins seen in chondrogenic murine ATDC5 or RMD-1 cells *in vitro*, where administration of transgenic IHH and SHH led to increased chondrogenic matrix formation [25]. In contrast to our study, however, Enomoto-Iwamoto and colleagues report on additive effects by treatment of IHH or SHH together with BMP-2 protein, which we attribute to the differences in protein delivery and target cell that were used in both experimental settings. This study also showed that IHH and SHH administration upregulated the expression of Ptc-1 and Gli-1 in RMD-1 and ATDC-5 cells, while Smo, BMP-2 and BMP-4 were unregulated but present and BMP-5 and BMP-7 were undetectable in these cell types [25], indicative of the major regulatory chondrogenic pathways that are also likely to be involved in our study using adult human MSCs.

Furthermore, experiments in SHH knockout mice with defective tracheal cartilage formation revealed that SOX9 was a downstream regulator of SHH, and that cartilage formation by exogenous administration with either SHH or BMP-4 could be rescued, indicating important roles for both factors on chondrogenesis [26]. This corresponds to *in vitro *data from ATDC-5 cells, where exogenously administered IHH upregulated the gene expression of COL type X and osteoprotegerin ligand, while it did not modulate the expression of IHH itself, BMP-4, BMP-6, TGF-β1, or TGF-β2 [27].

Nonetheless, there are also *in vivo *data on the formation of ectopic cartilage and bone in nude mice after intramuscular SHH and IHH administration [25], indicating the regenerative potential of such methodology. Moreover, Grande and colleagues were able to show improved cartilage regeneration *in vivo*, when genetically modified periosteal cells to express SHH or BMP-7 were administered orthotopically to osteochondral defects in a rabbit model [28]. Although no differences between both factors were delineated in this study, the issue of hypertrophy in the context of hedgehog factors was not further examined [28].

Much of the current knowledge about IHH is derived from research in the developing skeleton, where chondrocytes are condensed into cartilage elements during the process of endochondral ossification, and progress through stages of proliferation and hypertrophic differentiation, before they ultimately terminally differentiate, undergo apoptosis and are replaced by bone [29]. IHH has been shown to be involved as key regulator during several of these processes [14]. First, IHH is required for synthesis of PTHrP and interacts with PTHrP in a negative feedback loop regulating the pace of hypertrophic differentiation [30,31]. Apart from the action of IHH via PTHrP, further studies also indicate direct independent effects of IHH on chondrocyte proliferation and the ossification process, thus coordinating several different steps of endochondral bone formation [29,32]. Furthermore, the transcription factor Alf4 was shown to be required in chondrocytes for IHH expression, which subsequently, via the paracrine effects of IHH, dominates the role of Alf4 in osteoblasts during development for the control of early osteogenesis and skeletal growth [33]. Postnatally, the growth plate undergoes major structural and functional changes, with PTHrP being expressed in resting zone cartilage - a site that differs from the embryonic source, the periarticular cells - supporting the hypothesis that the embryonic IHH-PTHrP feedback loop is maintained in the postnatal growth plate [34]. These observations from the developing skeleton grossly correspond to our findings using adult MSCs, as IHH was shown be able to effectively induce and regulate several steps during chondrogenesis, including proliferation, differentiation, hypertrophy and apoptosis (Figures 2 to 7). Forced overexpression of IHH, however, might have resulted in an imbalance within the finely tuned regulative system with PTHrP that may have influenced the missing modulation of hypertrophy in our system. This is in contrast to the findings of Minina and colleagues in the cartilage elements of transgenic mice, where IHH overexpression resulted in a delayed onset of hypertrophic differentiation [35].

Notably, IHH only revealed the strongest staining of the matrix with COL type X and only very low levels of ALP activity, while most extensive staining for ALP was seen in the IHH + BMP-2 group and the BMP-2-only positive controls with only pericellular COL type X staining (Figure 5). This indicates that COL type X and ALP, both markers for chondrogenic hypertrophy, might be differentially regulated by IHH or BMP-2. This corresponds to findings of our own group and others in MSCs using TGF-β1 or BMP-2 [15,23], TGF-β1 or thyroid hormone [36], or BMP-4 or BMP-2 [11,37], where a similar differential regulation of these markers could also be observed. This is also in agreement with findings in the literature on different cell types, where COL type X precedes ALP expression in growth plate chondrocytes [38,39], costal cartilage [40] or MSCs [12].

This corresponds to elevated levels of COL type II and reduced levels of COL type X expression by TGF-β1-stimulated MSC aggregates upon stimulation with PTH hormone [41] or PTHrP *in vitro *[12,42]. Reduced levels of hypertrophy were also confirmed, when recombinant PTHrP was employed in an ectopic model of chondrogenesis *in vivo *using adult MSCs [12,42,43]. In our system, however, using adenoviral IHH gene delivery *in vitro*, no inhibitory effects on chondrogenic hypertrophy were evident. This pattern might change when IHH genes are delivered to cartilage defects *in vivo *- the clarifying experiments are underway.

Interestingly, during endochondral ossification, several other groups of signalling systems have been found to interact with IHH [29]. For example, several BMPs, such as BMP-2, are thought to act downstream of IHH, serving as secondary signals mediating the IHH signals to the periarticular perichondrium to induce PTHrP [29]. BMPs might also reciprocally act back on the prehypertrophic chondrocytes, thereby coordinating hypertrophic differentiation with the differentiation of the periosteum [29]. This is in agreement with reports of BMP-2 action on chondrogenesis and hypertrophy, which are in part regulated via HIF1α activation and vasculature formation [44]. This corresponds to our data, as highest levels of hypertrophy and apoptosis were seen in the group that co-expressed IHH and BMP-2 or BMP-2 alone, compared with the IHH + TGF-β1 or IHH only groups (Figures 5 to 8). This corresponds to the findings in several transgenic mouse models showing that, in particular, BMP-2 and TGF-β1 can have agonistic and antagonistic effects on proliferation and chondrogenesis and IHH regulation [45].

Moreover, IGF-1 seems also to be importantly involved in IHH/PTHrP signalling - as cartilage-specific IGF-1 receptor knockout mice revealed growth retardation with reduced chondrocyte proliferation and decreased IHH expression, while PTHrP was upregulated [33]. This involvement could also be confirmed *in vitro *using in IGF-1 receptor knockout in adult cells, indicating that the IGF-1 receptor in chondrocytes controls cell growth, survival, and differentiation in embryonic and postnatal growth in part by regulating the IHH/PTHrP pathway [33].

Finally, there are many more regulators of chondrocyte hypertrophy and terminal differentiation such as vitamin D3 [46,47] or fibroblast growth factor receptor-3 [29], which are also interacting with the IHH/PTHrP pathway - and it becomes more obvious that the single steps of endochondral ossification are tightly coordinated, which in the same way basically holds true for *in vitro *chondrogenesis and hypertrophy of adult MSCs. Although significant progress has been made during the last years in analysing the signals regulating endochondral ossification in the developing embryo, complete understanding of the control system in adult cells and tissues will require further extensive studies.

## Conclusion

IHH gene transfer via adenoviral vectors alone and in combination with TGF-β1 or BMP-2 efficiently induces chondrogenesis in aggregate cultures of human primary MSCs. The appearance of chondrocyte hypertrophy in this system following 3 weeks of *in vitro *culture could not be obviated by IHH, however, and was strongly present when BMP-2 was co-expressed. Whether IHH can induce chondrogenesis while modulating hypertrophy *in vivo *therefore remains to be seen. This gene transfer technology might thus be used to improve the outcome of MSC-based approaches to cartilage regeneration in humans in the future.

## Abbreviations

AGN: aggrecan core protein; ALP: alkaline phosphatase; Ad.: adenoviral vector; BMP: bone morphogenetic protein; COL: collagen; CS4: chondroitin-4-sulphate; COMP: cartilage oligomeric matrix protein; DMEM: Dulbecco's modified Eagle's medium; EF-1α: elongation factor-1α; ELISA: enzyme-linked immunosorbent assay; GAG: glycosaminoglycan; GFP: green fluorescent protein; H & E: haematoxylin and eosin; IGF-1: insulin-like growth factor-1; IHH: Indian hedgehog; MSC: mesenchymal stem cell; OP: osteopontin; PCR: polymerase chain reaction; PTHrP: parathyroid hormone-related peptide; RT: reverse transcription; SHH: sonic hedgehog; SOX9: sex determining region Y-box 9; TGF-β: transforming growth factor beta.

## Competing interests

The authors declare that they have no competing interests.

## Authors' contributions

All authors read and approved the manuscript and contributed to the study design, data analysis, interpretation of data, and drafting and revision of the manuscript. The data were generated by AFS, MK, SG and SCG, and a data review committee (AFS, SCG, SG, FG, FJ, UN and MR) analysed the data.
